# Exploring the clinical and genetic spectrum of Steel syndrome: two case reports and review of the literature

**DOI:** 10.3389/fmed.2026.1730466

**Published:** 2026-02-18

**Authors:** Daria Gorodilova, Vladimir Kenis, Khushnud Rustamov, Daria Akimova, Mikhail Skoblov, Elena Merkuryeva, Anna Morgul, Alyona Grigorieva, Liliia Andreeva, Victoria Zabnenkova, Maria Bulakh, Denis Chistol, Nasibakhon Raimkhodjaeva, Alexander Lavrov, Tatyana Hegay, Tamara Aripova, Sergey Kutsev, Tatiana Markova

**Affiliations:** 1 Research Centre for Medical Genetics, Moscow, Russia; 2 H.Turner National Medical Research Center for Children's Orthopedics and Trauma Surgery, Saint-Petersburg, Russia; 3 Republican Specialized Scientific and Practical Center for Traumatology and Orthopedics, Tashkent, Uzbekistan; 4 Department of Traumatology and Orthopedics, Saratov State Medical University named after V.I. Razumovsky, Saratov, Russia; 5 Saratov Regional Children's Clinical Hospital, Saratov, Russia; 6 Tashkent Research Center for Medical Genetics, Tashkent, Uzbekistan; 7 Immunogen Test LLC at Institute of Immunology and Human Genomics of the Academy of Science of the Republic of Uzbekistan, Tashkent, Uzbekistan; 8 Institute of Immunology and Human Genomics of the Academy of Science of the Republic of Uzbekistan, Tashkent, Uzbekistan

**Keywords:** COL27A1, phenotype, skeletal dysplasia, splicing assay, steel syndrome

## Abstract

Steel syndrome (STLS, OMIM# 615155) is a rare skeletal dysplasia associated with biallelic pathogenic variants in *COL27A1* gene. To date, more than 60 patients with STLS have been reported in the literature, the majority of whom are Puerto Rican. STLS in all individuals from this population is associated with the homozygous p.Gly697Arg missense variant, confirming the founder effect. Meanwhile, just 17 cases from 14 unrelated non-Puerto Rican families have been reported, including two fetuses. Here we present two pediatric cases of STLS from Russian and Uzbek populations, associated with two novel compound heterozygous splice variants c.2673 + 4A > G, c.2619 + 1G > A and a novel homozygous missense variant c.3988G > C p. (Gly1330Arg). Splicing assay was performed to investigate the novel donor splice site variants' effects on mRNA structure and expression. Both cases demonstrated skeletal features characteristic of STLS, including acetabular dysplasia or hip dislocation, carpal coalition, radial head dislocation, with additional extraskeletal manifestations observed in one patient. A review of 63 STLS cases revealed key diagnostic criteria present in the majority of individuals, though phenotypic variability was observed depending on variant type and population origin. It is proposed that biallelic Gly substitutions within the triple-helical domain associated with the ‘classic' skeletal phenotype of STLS initially characterized in Steel's study. In contrast, patients with homozygous or compound heterozygous frameshift or nonsense variants more frequently demonstrate a higher prevalence of extraskeletal manifestations and severe short stature. Our study expands the genetic and clinical spectrum of STLS in non-Puerto Rican populations and explores potential genotype-phenotype correlations, which will contribute to early disease diagnosis and the selection of optimal patient management strategies, avoiding unnecessary interventions.

## 1 Introduction

Steel syndrome (OMIM# 615155) is a rare autosomal recessive skeletal dysplasia, caused by biallelic pathogenic variants in *COL27A1* gene. The main clinical and radiological features include congenital hip and radial head dislocation, carpal coalition, acetabular dysplasia, short stature, and craniofacial dysmorphism. It was first delineated by Steel et al. in 1993, who reported a cohort of 23 Puerto Rican patients exhibiting similar clinical manifestations (1). Later, Flynn et al. described an additional 14 patients from the same population, and re-examined 18 subjects from Steel's cohort (1, 2). The presence of hip and radial head dislocations combined with joint laxity allowed for the inclusion of Larsen and Ehlers-Danlos syndromes in the differential diagnosis, while short stature suggested achondroplasia and pseudoachondroplasia. However, using targeted approaches to identify causative variants in disease-related genes, such as *COL5A1, COL1A1, COMP*, and *FGFR3*, did not yield any results (2). In 2015 Gonzaga-Jauregui et al. identified potential founder homozygous pathogenic variant c.2089G > C p. (Gly697Arg) in *COL27A1* causing Steel syndrome in a Puerto Rican family through exome sequencing (3). To date, approximately 66 patients with STLS have been reported in the literature, 51 of whom are of Puerto Rican origin (1, 2, 4). Additionally, two fetuses from one Caucasian family with suspected STLS were described by Frigola et al. (5). Molecular genetic testing was performed on a total of thirty-six individuals, including two fetuses. Among them eighteen were of Puerto Rican origin and one Hispanic, with all cases homozygous for the p.Gly697Arg variant, consistent with a founder effect (3, 6–8). In non-Puerto Rican patients twenty nucleotide variants were found, six of which resulted in Gly amino acid substitutions located in the triple-helical domain. Currently, two acceptor splice-site variants: c.3556 – 2A > G, c.4261 – 1G > A and one donor splice-site variant c.3249 + 1G > T have been reported in three unrelated families of Yemeni, Emirati, and Caucasian origin (5, 9, 10). Gradually accumulating data continue to expand the spectrum of not only radiological but also clinical manifestations of Steel syndrome, including various extraskeletal features such as sensorineural hearing loss, coloboma, genitourinary anomalies, and developmental delay (11–13).

Here, we report two pediatric cases of Steel syndrome: one associated with two novel compound heterozygous splice variants, and the other with a novel homozygous missense variant in *COL27A1* gene. We also explore potential genotype–phenotype correlations based on a review of clinical and radiological features in all reported cases with pathogenic variants in *COL27A1*.

## 2 Materials and methods

Blood samples were collected from the probands and their unaffected parents. Genomic DNA was extracted using standard methods. Whole-exome sequencing (WES) was used to perform DNA diagnosis in the probands. RT-PCR followed by deep sequencing was performed to analyze the mRNA structure of the two novel variants, using RNA extracted from the skin fibroblasts of proband 1 and his mother. Statistical analysis was performed to evaluate the prevalence and significance of clinical and radiological features reported in patients with Steel syndrome. Detailed description of the clinical, genetic, functional, and statistical methods used to analyze reported patients with Steel syndrome is provided in Supplementary Material 1.

## 3 Clinical reports

A detailed clinical and radiological data were obtained from two unrelated patients. All participants gave informed consent to the clinical and radiological examination and the publication of their anonymized data.

### 3.1 Family 1

A 3-year-old boy (Proband 1; P1), the only child in the family, was referred to a geneticist due to short stature, lower limb deformities, and progressive scoliosis. His parents were healthy, non-consanguineous individuals of Russian origin. During this first pregnancy, a prenatal ultrasound at 28 weeks revealed placental insufficiency, oligohydramnios, and intrauterine growth restriction grade 2–3. A preterm delivery was performed at 33 weeks and 4 days, by emergency cesarean section due to the progression of fetoplacental insufficiency. Birth weight was 1720 g (−1.44 SD), the length – 41 cm (−2.04 SD), and the Apgar score was 4/5. Immediately after birth, he was admitted to the intensive care unit due to respiratory failure and depressed consciousness, and was placed on mechanical ventilation for 2 days. During the early orthopedic examination, the following clinical and radiological features were observed: sternocleidomastoid muscle injury, right-sided torticollis, C3 vertebral instability, wedge-shaped deformity of C3 and C4 vertebrae with cervical kyphosis, mild acetabular dysplasia, congenital scoliosis, planovalgus deformity of the left foot and equinovarus deformity of the right foot. Additionally, bilateral cryptorchidism was detected. Independent walking was achieved at 21 months, indicating delayed motor development. By 3 years, the child had achieved phrasal speech, consistent with mild speech delay. At 3.5 years bilateral sensorineural hearing loss (SNHL) grade 2–3 was diagnosed.

**Figure 1:**
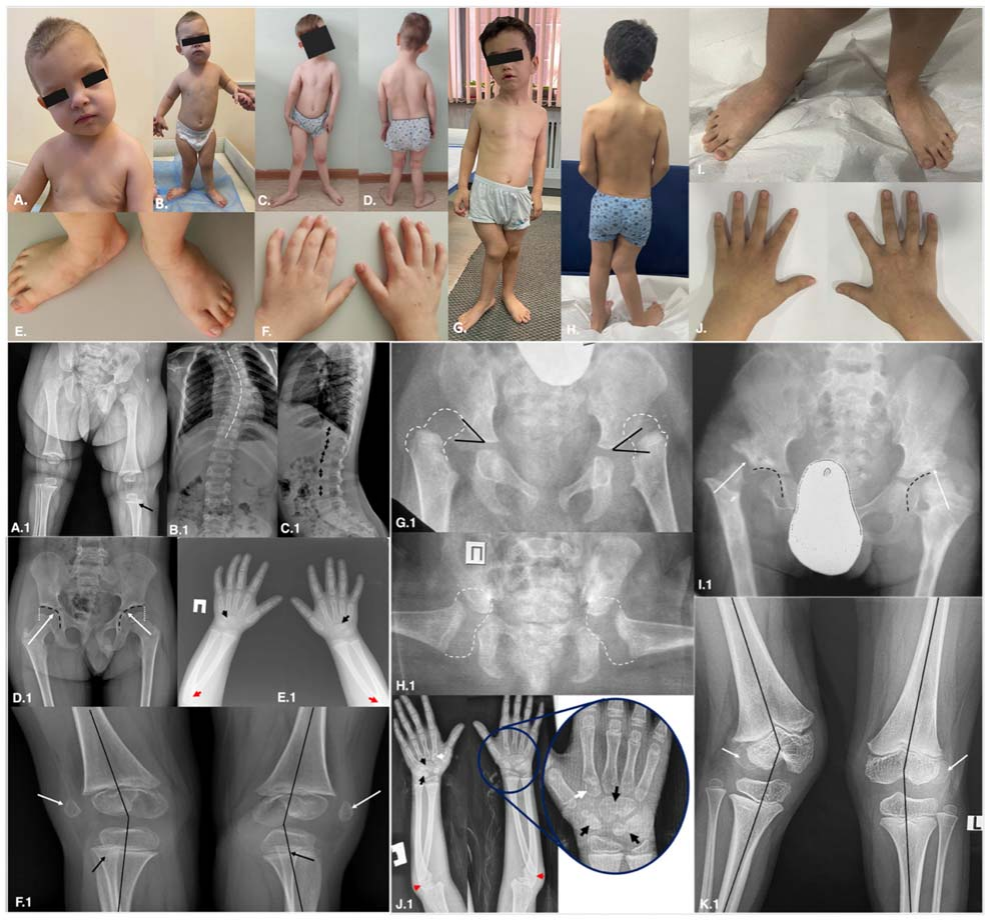
P1 phenotype at the age of 3.6 years **(A, B)** and at 5 years **(C–F)**. P2 phenotype at the age of 7 years **(G-J)**. Prominent forehead, hypertelorism, mild rhizomelic limb shortening, mild pectus excavatum, bilateral 5^th^ finger clinodactyly, genu valgum and mild partial cutaneous syndactyly of 2^nd^ and 3^rd^ toes were observed in both probands. P1 radiological findings at 10 months **(A.1)** and 5 years **(B.1–F.1)**. P2 radiographs at 1 year **(G.1;H.1)** and at 6 years **(I.1–K.1)**. **(A.1)** Normally centered hip joints with mild acetabular dysplasia, rotational subluxation of the knee presented with dorsally displaced proximal fibula (black arrow). **(B.1;C.1)** Severe thoracic scoliosis (white broken line), ‘tall' vertebral bodies (black arrows). **(D.1)** Normally centered hip joints with mild acetabular dysplasia (white arrows) with mild undercoverage of the femoral heads (white dotted lines). **(E.1)** Radial head dislocations (red arrows), multiple fusions of the carpal bones (black arrows). **(F.1)** Valgus knee deformity (black lines), lateral dislocation of the patella (white arrows), lateral and rotational subluxation of the tibiae. **(G.1)** Bilateral hip dislocation, dysplastic acetabulae (acetabular index 45° marked with the black lines). **(H.1)** Frog-leg position under the general anesthesia during an attempt of close reduction demonstrates irreducibility of the dislocations; non-ossified proximal femora marked with white broken lines. **(I.1)** Radiograph of the hips after surgery: residual hip dislocation, deformed proximal femoral neck and head oriented onto the supraacetabular region (white arrows); acetabular cavities are empty (marked with black broken lines). **(J.1)** Radial head dislocations (red arrows), multiple fusions of the carpal bones (black arrows), abnormal ossification pattern of the second metacarpals – pseudoepiphysis of the base of the second metacarpal bones (white arrows). **(K.1)** Valgus knee deformity (black lines); lateral dislocation of the patella (white arrows), lateral and rotational subluxation of the tibiae.

Upon examination by a geneticist at 3 years 8 months, characteristic facial dysmorphism was observed, including oval-shaped face with asymmetry, prominent forehead, hypertelorism, broad nasal bridge, micrognathia. His height was 90 cm (−2.4 SD), weight – 14.1 kg (– 0.99 SD) and head circumference – 46 cm (−2.61 SD). Further phenotypic features included kyphoscoliosis, nipple hypertelorism, restriction of elbow extension and right-sided equinovarus deformity (Figure 1). At the follow-up examination at 5 years, his height was 100 cm (– 1.75 SD), indicating the absence of severe growth retardation.

### 3.2 Family 2

A 7-year-old boy (Proband 2;P2) was referred to a geneticist due to lower limb deformities, gait abnormalities, and congenital hip dislocation that had not improved with conservative or surgical approaches. He is the first child of healthy, third-generation consanguineous parents of Uzbek origin (Supplementary Material 2 Figure S2). There are also two healthy siblings – a boy and a girl, 3 and 1.5 years old, respectively. It was the first delivery at term. The birth weight was 3600 g (0.07 SD), the length – 50 cm (−0.38 SD), and the Apgar score was 7/8. Shortly after birth left-sided torticollis, acetabular dysplasia and congenital hip dislocation were identified. Conservative management with splinting was initiated at six months of age. At 2 years, skeletal traction without significant positive outcome was performed. Hip arthroplasty was subsequently performed at ages two, three, and four, however, neither procedure resulted in substantial clinical benefit. His motor and cognitive development were age-appropriate. However, independent walking was achieved only at 4 years due to orthopedic limitations. At the age of 6, first-degree conductive hearing loss was diagnosed, linked to severe adenoid hypertrophy. At 7 years, his height was 111 cm (−1.74 SD), weight – 18 kg (−2.01 SD) and head circumference – 50.5 cm (−0.52 SD). The observed facial phenotype includes an oval-shaped face, a prominent forehead, hypertelorism, hypoplasia of the midface, broad nasal bridge and a cup-shaped right auricle with hypoplasia of the helix. Additional features included mild left-sided torticollis and scapular asymmetry, pectus excavatum, nipple hypertelorism, scoliosis, pelvic torsion, genu valgum more prominent on the right-side, bilateral shortening of the 4^th^ and 5^th^ toes, pes planovalgus. Significant hypermobility of the interphalangeal joints, as well as contractures in the elbow and knee joints, were also observed (Figure 1).

### 3.3 Molecular and splicing analyses

WES was performed on P1 and P2 due to their phenotypic features, which were suggestive of rare skeletal dysplasia. In P1, two heterozygous novel splice variants were identified in *COL27A1* (NM_032888.4): c.2619 + 1G > A in intron 17 and c.2673 + 4A > G in intron 18. Segregation analysis by Sanger sequencing confirmed their inheritance from the parents. The c.2619 + 1G > A variant was classified as pathogenic (PM2, PVS1, PP3) according to ACMG guidelines, while the c.2673 + 4A > G variant was categorized as a variant of uncertain significance (PM2, PP3) (14).

In P2, novel homozygous missense variant c.3988G > C p. (Gly1330Arg) was identified in *COL27A1*. This variant is present in population controls in gnomAD (v4.1.0) with a frequency of 6.212e-7. The AlphaMissense score is 0.972 classifying as likely pathogenic (15). Segregation analysis by Sanger sequencing confirmed its inheritance from the parents. This variant was classified as likely pathogenic (PM1, PM2, PP3, PP2) according to ACMG guidelines (14).

To investigate the variants' effects on mRNA structure and expression we developed an RT-PCR system targeting the mRNA locus spanning exons 16 to 19 and including both nucleotide variants. RT-PCR analysis of a control sample followed by deep targeted sequencing revealed two alternative splicing events between exons 16 and 17: one producing the reference isoform and another generating an alternative transcript due to the activation of a cryptic donor splice site in exon 16, resulting in an 8-nucleotide truncation of exon 16. This isoform, designated AB058773 and recorded in the GenBank database, lacks a complete open reading frame (ORF) due to an 8-nt truncation and is predicted to be non-functional based on premature termination.

For the c.2619 + 1G > A variant, a bioinformatic analysis was first conducted using the SpliceAI predictor (v1.3.1), which predicted disruption of both the acceptor and donor splice sites of exon 17 (delta scores 0.81 and 0.91, respectively). Subsequent RT-PCR analysis of total RNA from the proband's blood samples revealed not only the two expected reference isoforms (similar to controls) but also a significant proportion of aberrant transcripts with exon 17 skipped. *In silico* predictions supported these findings, indicating that c.2619 + 1G > A causes exon 17 skipping, resulting in an in-frame deletion of 18 amino acids p. (Gly856_Lys873del).

**Figure 2:**
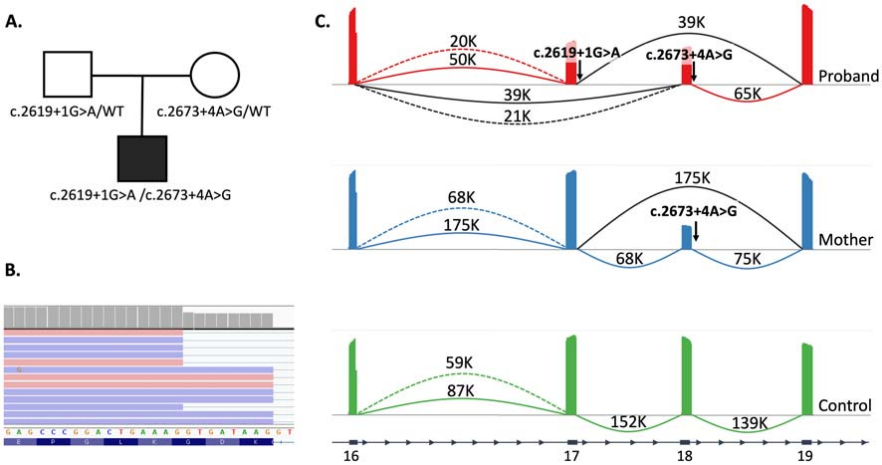
**Splicing assay performed in Proband 1. (A)** Pedigree of the P1 family. **(B)** The inclusion of both the canonical and cryptic donor splice sites of exon 16 leads to alternative splicing, which was detected in all three studied samples. **(C)** The sashimi plot illustrates the results of deep targeted RNA sequencing performed on fibroblast cultures (proband, mother, control). However, the father's skin fibroblasts were unavailable for analysis. The proband showed exon 17 skipping on one allele and exon 18 skipping on the other. The maternal sample exhibited an aberrant transcript lacking exon 18 in addition to the wild-type *COL27A1* mRNA.

Bioinformatic analysis of the c.2673 + 4A > G variant suggested a potential effect on the canonical splice sites of exon 18 (delta scores 0.34 and 0.37). In this case, RNA analysis was performed on mRNA samples isolated from the proband's and his mother's fibroblasts. Deep sequencing data visualized in Sashimi plots confirmed the presence of both two reference isoforms and an aberrant transcript with exon 18 skipped (Figure 2). Based on the bioinformatic analysis, we conclude that c.2673 + 4A > G leads to exon 18 skipping, producing another 18-amino acid deletion p.Gly874_Leu891del. Both variants detected in the proband cause splicing defects, likely contributing to the phenotype. This evidence supported reclassification of variant c.2673 + 4A > G as likely pathogenic (PM1, PM2, PM4, PP3) (14).

## 4 Discussion

We report two novel cases of Steel syndrome in pediatric patients of Russian and Uzbek origin. The clinical manifestation of STLS in our patients included hallmark features such as acetabular dysplasia or congenital hip dislocation, radial head dislocation, carpal coalition and facial dysmorphism. In P1, the disease was associated with two novel splice variants in the *COL27A1* – c.2619 + 1G > A and c.2673 + 4A > G, causing skipping of exons 17 and 18, confirmed by RT-PCR followed by deep sequencing. In P2 a homozygous missense variant c.3988G > C p. (Gly1330Arg) located in the triple-helical domain of the protein was identified.

*COL27A*1 gene is located on human chromosome 9q32–33 and consists of 61 exons. The proα1(XXVII) protein, encoded by this gene, is 1860 amino acids long and like other proteins belonging to this large family contains a triple-helical domain composed of repeating Gly-Xaa-Yaa motifs (16). It is significantly expressed in cartilage, eye, inner ear structures, bronchial and lung epithelium, ameloblasts in developing teeth, as well as major heart arteries during early embryonic development, which may explain the presence of extraskeletal features such as sensorineural hearing loss, crowded teeth, septal defects, and partial syndactyly in patients with STLS (6, 16). However, in later developmental stages and throughout adulthood, its expression remains predominantly restricted to cartilage, especially within the growth plate, which undergoes constant renewal during endochondral bone growth (16–18).

The majority of patients with STLS reported to date are of Puerto Rican origin. Among them, 19 individuals, including one patient of Hispanic origin, were found to have the homozygous variant p.Gly697Arg in the *COL27A1* (3, 6–8). This Gly amino acid substitution is located in the triple-helical domain of the protein (6, 16). Interestingly, most diseases associated with Gly amino acid substitutions within this domain of other collagens follow an autosomal dominant mode of inheritance, demonstrating a dominant-negative effect by impairing proper protein folding and leading to endoplasmic reticulum stress (19, 20). This results from the fact that only glycine is small enough to fit into the center of the assembled triple-helical structure without disrupting its stability. Therefore, any deviation from the conserved glycine residues in vertebrate fibrillar collagens often leads to structural instability and disease (16). However, COL27A1 as a member of type C clade of vertebrate fibrillar collagens, along with COL24A1, may be more resistant to disruptions of the Gly-Xaa-Yaa motif particularly regarding folding and secretion, which suggests that this mechanism is unlikely to play a significant role in the development of COL27A1– related skeletal dysplasia (6, 17, 21).

**Figure 3:**
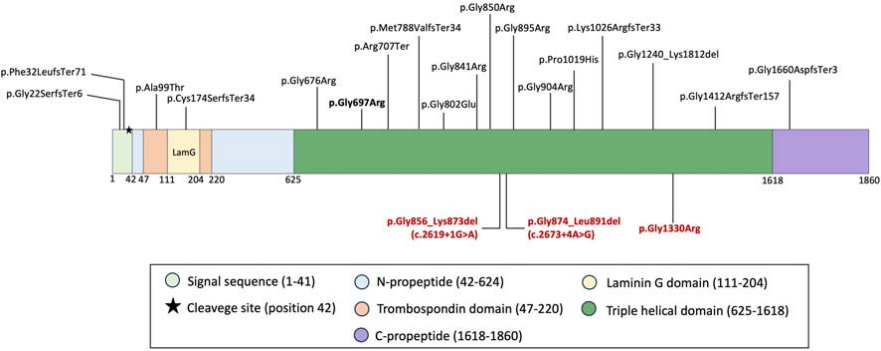
Schematic representation of COL27A1 protein domains [adapted from Pace et al., (16) and Girisha et al., (4)]. Domain distribution of all reported and three novel nucleotide variants identified in STLS patients [excluding splice-site variants: c.3249 + 1G > T, c.3556-2A > G and c.4261 – 1G > A reported by Frigola et al., (5); Gariballa et al., (9) and Maddirevula et al., (10)]. Novel variants identified in P1 and P2 are highlighted in red and bold font. The founder allele, prevalent in individuals of Puerto Rican origin, is indicated in bold and black font.

To date, twenty one pathogenic nucleotide variants distributed throughout the *COL27A1* gene have been reported (Figure 3; Supplementary Material 2 Table S4) (3, 4, 6, 9–13, 22–24).

Approximately half of the reported variants are frameshift or nonsense, likely leading to protein truncation, consequent degradation and loss of function. The variant c.3556 – 2A > G identified in the Emirati patient with STLS was shown to disrupt splicing by activating alternative cryptic splice sites. This resulted in the production of three distinct transcripts, each leading to a different deletion. Based on these findings, the authors suggested that the truncated protein may lead to aberrant folding and could be targeted for subsequent degradation, that may be more damaging than previously reported missense variants (9). Indeed, a 9-year-old Syrian patient with compound heterozygous variants p.Phe32LeufsTer71 and p.Lys1026ArgfsTer33 exhibited profound short stature (85.5 cm, – 7.9 SD) along with extraskeletal features including early-onset SNHL, iris coloboma, and developmental delay (13). While, among all individuals with normal (less than −2 SD) or slightly decreased height, missense variants, typically homozygous Gly substitutions in the triple-helical domain were detected, except for two sibs from Satoh et al. study, where the compound heterozygous variants p.Gly676Arg and p.Met788ValfsTer34 were identified (Supplementary Material 2 Tables S1–S2) (8, 11, 22). However, the presence of a frameshift variant on one of their alleles may have contributed to their clinical presentation. STLS phenotype observed in P1 was associated with in-frame deletions p.Gly874_Leu891del and p.Gly856_Lys873del, located within the triple helical domain. Disease presentation in P1 was characterized by the absence of short stature (−1.75 SD) and hip dislocation combined with extraskeletal features (Table 1). This suggests that in-frame deletions likely cause misfolding, leading to a less profound functional defect than truncating variants such as frameshift or nonsense, possibly explaining the milder skeletal phenotype and supporting the idea that COL27A1 may be more resistant to disruptions of the Gly-Xaa-Yaa motif (6, 17, 21). Since precise genotype-phenotype correlations remain undefined in Steel syndrome, we conducted a comprehensive review of clinical and radiological manifestations in all reported cases (Table 1).

**Table 1:** Summarized data of STLS clinical and radiological features observed in patients from the literature and the present study (*n* = 63).

**Criteria**	**Puerto – Rican patients from Flynn et al. (2) (*n* = 32)**	**Puerto – Rican patients (other studies; *n* = 14)**	**Non – Puerto – Rican patients (*n* = 15)**	**Present study P1**	**Present study P2**	**Total (*n* = 63)**
Min and max age	3.6–50 y (average 13.7)	2 m−74 y	1.3 – 11 y	5	7	–
Sex	f (*n* = 15); m (*n* = 17)	f (*n* = 8); m (*n* = 6)	f (*n* = 5) m (*n* = 10)	m	m	f (*n* = 28) m (*n* = 35)
Short stature	32/32	12/14	11/13	–	–	55/61
Normal stature	0/32	2/14	2/13	+	+	6/61
Pectus excavatum	n/d	2/3	6/6	+	+	10/11
Limb shortening	n/d	n/d	9/12	+	+	11/14
**Facial features**						
Oval-shaped face	32/32	2/2	8/12	+	+	44/48
Prominent forehead and/or frontal bossing	32/32	3/5	13/14	+	+	50/53
Hypertelorism	32/32	3/5	9/11	+	+	46/50
Midface hypoplasia	n/d	4/5	14/14	–	+	19/21
Long philtrum	n/d	2/2	9/11	–	–	11/13
**Radiological features**						
Congenital hip dislocation	32/32	9/14	11/14	_	+	53/62
Poorly ossified femoral head	n/d	3/5	9/9	_	+	13/16
Acetabular abnormalities	n/d	3/5	10/10	+	+	15/17
Carpal coalition	29/32	4/7	3/11	+	+	38/52
Radial head dislocation	29/32	8/14	12/13	+	+	51/61
Scoliosis	17/32	10/14	8/14	+	+	37/62
Cervical spine abnormalities	3/32	7/8	1/5	+	–	12/47
Lumbar lordosis	10/32	5/10	4/10	–	–	19/52
Coxa vara	n/d	1/2	8/11	–	–	9/13
Genu valgum	n/d	n/d	9/12	+	+	11/12
Genu varum	n/d	n/d	3/12	–	–	3/14
Patellar dislocation	n/d	n/d	5/5	+	+	7/7
**Upper limbs abnormalities**						
Partial cutaneous syndactyly of fingers or/and toes	n/d	n/d	7/7	+	+	9/9
Bilateral 5^th^ finger clinodactyly	n/d	2/2	10/10	+	+	14/14
**Lower limbs abnormalities**						
Pes cavus	11/32	0/14	2/11	–	–	13/59
Various foot deformity	0/32	5/14	6/11	Pes planovalgus Pes equinovarus	Pes planovalgus	13/59
Vertical talus	1/32	0/2	5/6	–	–	6/42
**Extraskeletal features**						
Cryptorchidism	n/d	n/d	6/7	+	–	7/9
Inguinal or umblical hernia	n/d	n/d	5/6	–	–	5/8
Developmental delay	n/d	1/8	8/13	+	–	10/23
Hearing loss	0/32	4/9 (unspecified *n* = 4)	13/14 (SNHL *n* = 4; unspecified *n* = 9)	+ SNHL	+ (conductive^a^)	18/56
Motor delay	n/d	3/5	3/10	+	–	7/17
Delayed speech	n/d	n/d	6/10	+	–	7/12
Other	Tarsal coalition (*n* = 1)	Table S3	Table S1 and S2	OD: mixed astigmatism; OS: compound myopic astigmatism;	See the article content	–

(+): Present; (–): Absent; n/d – no data. The features included in the table were selected based on analysis of clinical cases reported in the literature. However, the lack of information about presence or absence of certain features in patient descriptions may affect interpretation.

^a^Cases of conductive hearing loss unrelated to STLS were omitted from the feature frequency analysis.

Phenotypic analysis of STLS cases revealed features that appear to be characteristic of particular patient subgroups. Interestingly, pes cavus deformity and carpal coalition are most common in individuals of Puerto Rican origin, while equinovarus or planovalgus deformities and hearing loss are more often noted in other populations (Table 1) (4, 6, 11, 23). Furthermore, certain features, such as cervical spine abnormalities and associated complications such as cervical stenosis and cervical cord compression, likely manifest only during adulthood (2, 6, 7).

Recently, it was suggested that hearing loss in individuals with STLS associated with the homozygous founder allele p.Gly697Arg may occur later, after the age of 30. In contrast, individuals with more ‘severe' alleles experience the early-onset hearing loss (6). Indeed, three Puerto Rican individuals homozygous for the p.Gly697Arg variant were diagnosed with hearing loss in adulthood (6). According to reported data, 7 out of 16 patients with early-onset hearing loss carried variants predicted to result in premature translation termination and possible loss of function. Two individuals, including P2, were diagnosed with conductive hearing loss unrelated to STLS (6). Patients with the homozygous p.Gly802Glu, p.Gly904Arg, and p.Gly841Arg variants, as well as P1 with in-frame deletions within the triple helical domain, were diagnosed with early-onset hearing loss (Supplementary Material 2 Tables S1–S3). These variants are predicted to cause protein misfolding. Early-onset hearing loss was also diagnosed in three additional patients: two with compound heterozygous variants p.Ala99Thr and p.Pro1019His and one with a homozygous splice variant c.3556 – 2A > G (4, 9). The analysis indicates that the age of hearing loss onset is not correlated with the location or type of genetic variant. However, this hypothesis requires further validation through the accumulation of additional clinical patient data.

Notably, P1 presented with extraskeletal manifestations, including SNHL, mild developmental delay, and cryptorchidism possibly associated with prematurity, but did not exhibit the classic diagnostic criteria of Steel syndrome—congenital hip dislocation and short stature (Figure 1). Despite initial data on obligatory presence of hip dislocation, there is more evidence of less specific importance of this condition as a part of the syndrome. Thus, in both the original Steel et al. study and the subsequent Flynn et al. study hip dislocations were a universal finding (Table 1) (1, 2). Surprisingly, the first European patient reported by Kritioti et al. had no hip dislocations but only minor stable hip changes, radiographically similar to our P1 case (22). Kim et al. reported a Korean patient presented with bilateral hip dysplasia and pointed out that hip abnormalities in STLS may vary from acetabular dysplasia to complete dislocation (12). These observations suggest that the ‘classic' phenotype may be predominantly linked to Gly substitutions in the triple helical domain. This is supported by our findings, particularly in P2, who exhibited the complete spectrum of STLS criteria as defined by Steel et al., while lacking extraskeletal manifestations typically observed in non-Puerto Rican individuals (Table 1) (1). Hand radiographs demonstrated abnormal ossification patterns characterized by carpal bone coalescence in both patients. While the whole extent of fusion was challenging to evaluate due to the early ossification stage, typical capitate-hamate coalition was easily recognizable in both patients. One more interesting finding in P2 was pseudoepiphyses of the second metacarpals (Figure 1). Multiple pseudoepiphyses of the metacarpals and metatarsals may be seen in otherwise healthy children but can also occur in patients with skeletal dysplasias (25). Radiographic analysis demonstrated that our patients exhibited the same ossification pattern as the Indian monozygotic twins with STLS reported by Girisha et al. in 2022, but this phenomenon was not mentioned (4).

A comprehensive review of symptoms prevalence across reported patients including P1 and P2 from present study highlighted core features occurring more frequent among all STLS patients such as short stature, congenital hip and radial dislocation, carpal coalition and facial dysmorphism (Supplementary Material 2 Figure S1). These symptoms represent a core cluster of STLS skeletal features that may facilitate early diagnostic suspicion and help to avoid unnecessary surgical procedures for hip dislocation correction (2). However, comparison across Puerto Rican patients and individuals from other populations revealed phenotypic variability, including missing hallmark features like short stature or hip dislocation and increased incidence of extraskeletal manifestations, which should also be considered (Table 1).

In conclusion, we report two pediatric cases of STLS associated with novel biallelic variants in the *COL27A1*. This study provides the first characterization of two splice donor site variants confirming their effect on splicing. Review of reported clinical data enabled identification of a phenotypic cluster characteristic of STLS. Furthermore, our findings indicate that the presence of protein-truncating variants may be associated with more severe disease course characterized by extraskeletal manifestations, developmental delay and pronounced short stature. In contrast, missense variants, particularly glycine substitutions within the triple-helical domain, are more often associated with ‘classic' phenotype initially reported by Steel et al. (1). To date, most reported cases have involved Puerto Rican patients with a homozygous founder allele. Therefore, expanding the genetic and phenotypic spectrum of STLS in diverse populations and age groups will be essential for improving diagnostic accuracy and clinical management.

## Data Availability

The original contributions presented in the study are included in the article/Supplementary material, further inquiries can be directed to the corresponding author.

## References

[1] Steel HH Piston RW Clancy M Betz RR. A syndrome of dislocated hips and radial heads, carpal coalition, and short stature in Puerto Rican children. J Bone Joint Surg Am. (1993) 75:259–64 doi: 10.2106/00004623-199302000-00013 8423186

[2] Flynn JM Ramirez N Betz R Mulcahey MJ Pino F Herrera-Soto JA . Steel syndrome: Dislocated hips and radial heads, carpal coalition, scoliosis, short stature, and characteristic facial features. J Pediatr Orthop. (2010) 30:282–88. doi: 10.1097/BPO.0b013e3181d3e464 20357596

[3] Gonzaga-Jauregui C Gamble CN Yuan B Penney S Jhangiani S Muzny DM . Mutations in COL27A1 cause Steel syndrome and suggest a founder mutation effect in the Puerto Rican population. Eur J Hum Genet. (2015) 23:342–46. doi: 10.1038/ejhg.2014.107 24986830 PMC4326704

[4] Girisha KM Jacob P SriLakshmi Bhavani G Shah H Mortier GR. Steel syndrome: report of three patients, including monozygotic twins and review of clinical and mutation profiles. Eur J Med Genet. (2022) 65:104521 doi: 10.1016/j.ejmg.2022.104521 35568358

[5] Frigola G Del Rincón OG Florián VB Fita AV Campos B Pauta M . Histopathology of recurrent Steel syndrome in fetuses caused by novel variants of COL27A1 gene. Virchows Arch. (2021) 479:413–18. doi: 10.1007/s00428-020-02979-2 33411029

[6] Gonzaga-Jauregui C Yesil G Nistala H Gezdirici A Bayram Y Nannuru KC . Functional biology of the Steel syndrome founder allele and evidence for clan genomics derivation of COL27A1 pathogenic alleles worldwide. Eur J. Hum Genet. 2020 289 (2020) 28:1243–64 doi: 10.1038/s41431-020-0632-x 32376988 PMC7608441

[7] Belbin GM Odgis J Sorokin EP Yee MC Kohli S Glicksberg BS . Genetic identification of a common collagen disease in puerto ricans via identity-by-descent mapping in a health system. Elife. (2017) 6:e25060 doi: 10.7554/eLife.25060 28895531 PMC5595434

[8] Amlie-Wolf L Moyer-Harasink S Carr AM Giampietro P Schneider A Simon M. Three new patients with steel syndrome and a puerto rican specific COL27A1 mutation. Am J Med Genet Part A. (2020) 182:798–803. doi: 10.1002/ajmg.a.61465 31903681

[9] Gariballa N Ben-Mahmoud A Komara M Al-Shamsi AM John A Ali BR . Novel aberrant splice site mutation in COL27A1 is responsible for Steel syndrome and extension of the phenotype to include hearing loss. Am J Med Genet Part A. (2017) 173:1257–63. doi: 10.1002/ajmg.a.38153 28322503

[10] Maddirevula S Alzahrani F Al-Owain M Al Muhaizea MA Kayyali HR AlHashem A . Autozygome and high throughput confirmation of disease genes candidacy. Genet Med. (2019) 21:736–42. doi: 10.1038/s41436-018-0138-x 30237576 PMC6752307

[11] Satoh C Kondoh T Shimizu H Kinoshita A Mishima H Nishimura G . Ichiro Brothers with novel compound heterozygous mutations in COL27A1 causing dental and genital abnormalities. Eur J Med Genet. (2021) 64:104125. doi: 10.1016/j.ejmg.2020.104125 33359165

[12] Kim JS Jeon H Lee H Ko JM Kim Y Choi M . Biallelic novel mutations of the COL27A1 gene in a patient with Steel syndrome. Hum Genome Var. (2021) 8:1–4. doi: 10.1038/s41439-021-00149-7 33963180 PMC8105406

[13] Pölsler L Schatz UA Simma B Zschocke J Rudnik-Schöneborn S. A Syrian patient with Steel syndrome due to compound heterozygous COL27A1 mutations with colobomata of the eye. Am J Med Genet Part A. (2020) 182:730–34. doi: 10.1002/ajmg.a.61478 31913554 PMC7079147

[14] Richards S Aziz N Bale S Bick D Das S Gastier-Foster J . Standards and guidelines for the interpretation of sequence variants: a joint consensus recommendation of the American College of Medical Genetics and Genomics and the Association for Molecular Pathology. Genet Med. (2015) 17:405–24. doi: 10.1038/gim.2015.30 25741868 PMC4544753

[15] Cheng J Novati G Pan J Bycroft C Žemgulyte A Applebaum T . Accurate proteome-wide missense variant effect prediction with AlphaMissense. Science. (2023) 381:eadg7492 doi: 10.1126/science.adg7492 37733863

[16] Pace JM Corrado M Missero C Byers PH. Identification, characterization and expression analysis of a new fibrillar collagen gene, COL27A1. Matrix Biol. (2003) 22:3–14. doi: 10.1016/S0945-053X(03)00007-6 12714037

[17] Plumb DA Ferrara L Torbica T Knowles L Mironov A Kadler KE . Collagen XXVII organises the pericellular matrix in the growth plate. PLoS ONE. (2011) 6:e29422. doi doi: 10.1371/journal.pone.0029422 22206015 PMC3242791

[18] Plumb DA Dhir V Mironov A Ferrara L Poulsom R Kadler KE . Collagen XXVII is developmentally-regulated and forms thin fibrillar structures distinct from those of classical vertebrate fibrillar collagens. J Biol Chem. (2007) 282:12791. doi: 10.1074/jbc.C700021200 17331945 PMC2688011

[19] Gawron K. Endoplasmic reticulum stress in chondrodysplasias caused by mutations in collagen types II and X. Cell Stress Chaperones. (2016) 21:943–58. doi: 10.1007/s12192-016-0719-z 27523816 PMC5083666

[20] Viakhireva I Bychkov I Markova T Shatokhina O Karandasheva K Udalova V . The molecular complexity of COL2A1 splicing variants and their significance in phenotype severity. Bone. (2024) 181:117013. doi: 10.1016/j.bone.2024.117013 38246255

[21] Boot-Handford RP Tuckwell DS Plumb DA Farrington Rock C Poulsom R. A novel and highly conserved collagen (proα1(XXVII)) with a unique expression pattern and unusual molecular characteristics establishes a new clade within the vertebrate fibrillar collagen family. J Biol Chem. (2003) 278:31067–77. doi: 10.1074/jbc.M212889200 12766169

[22] Evie Kritioti Athina Theodosiou Nayia Nicolaou Angelos Alexandrou Ioannis Papaevripidou Elisavet Efstathiou . First reported case of Steel syndrome in the European population: A novel homozygous mutation in COL27A1 and review of the literature. Eur J Med Genet. (2020):63 doi: 10.1016/j.ejmg.2020.103939 32360765

[23] Kotabagi S Shah H Shukla A Girisha KM. Second family provides further evidence for causation of steel syndrome by biallelic mutations in COL27A1. Clin Genet. (2017) 92:323–26. doi: 10.1111/cge.13006 28276056

[24] Thuresson AC Soussi Zander C Zhao JJ Halvardson J Maqbool K Månsson E . Whole genome sequencing of consanguineous families reveals novel pathogenic variants in intellectual disability. Clin Genet. (2019) 95:436–39. doi: 10.1111/cge.13470 30525197 PMC6392105

[25] Markova TV Kenis VM Melchenko EV Demina NA Gundorova P Nagornova TS . Clinical and genetic characteristics and orthopedic manifestations of the Saul–Wilson syndrome in two Russian patients. Pediatr Traumatol Orthop Reconstr Surg. (2020) 8:451–60. doi: 10.17816/PTORS33826

