# Supplementary material for: Exploring the clinical and genetic spectrum of Steel syndrome: two case reports and review of the literature
Source: Front Med (Lausanne). 2026 Feb 18;13:1730466. doi: 10.3389/fmed.2026.1730466 (PMC12956644; doi:10.3389/fmed.2026.1730466)
Supplement: Supplementary file 1 [file Supplementary_file_1.docx]

Supplementary Material 1

**Materials and Methods**

*Whole exome and Sanger sequencing*

For molecular genetic studies, blood samples were collected from the affected individuals and healthy family members. WES was performed on the DNBSEQ-400 platform (MGI, China). DNA was extracted from blood using the QIAamp DNA Mini Kit (QIAGEN, Netherlands) and quantified with the Qubit dsDNA Assay Kit (Thermo Fisher Scientific, USA). Libraries were prepared using the Nanodigmbio NadPrep DNA Library Kit (for MGI) (Nanodigmbio, China) and assessed for size distribution on the Agilent 4200 TapeStation (D1000 HS ScreenTape, Agilent, Santa Clara, CA, USA). Target enrichment was performed with the Nanodigmbio NEXome Plus Panel v1.0 (Nanodigmbio, China), covering coding regions of 20,000 genes. The PCR product was circularized, and 60 fmol of ssCirDNA was amplified via rolling-circle amplification to generate DNA nanoball libraries, which were sequenced on the DNBSEQ-400 (paired-end, 150 bp reads). The average coverage of the whole exome in patients was over ×150; the percentage of target regions with coverage ≥×10 was 98%. Variant annotation was performed using the nomenclature available at http://varnomen.hgvs.org/recommendations/DNA, version 21.1.1. The sequencing data were analyzed using the «NGS-data-Genome» software (https://ngs-data-ccu.epigenetic.ru/main/, accessed on 20 July 2025), created by the Bioinformatics Department of the Research Centre for Medical Genetics, Russia (registration number № 2021662119)[1]. Population frequencies and clinical relevance of identified variants were assessed using data from the 1000 Genomes Project, ESP6500, and Genome Aggregation Database (gnomAD) v3.1.2. The clinical significance of the variants was evaluated using ACMG criteria for variant interpretation[2]. To validate the identified nucleotide variants in the affected patient and their family members, Sanger sequencing was performed using the ABI Prism 3500xl Genetic Analyzer (Thermo Fisher Scientific, Waltham, MA, USA). Primer sequences were selected according to the reference sequence of the target regions of the *COL27A1* (NM_032888.4).

*RNA analysis*

The mRNA structure analysis was performed using RNA extracted from skin fibroblasts of the proband and the proband’s mother. Both cell lines were obtained from the Moscow Branch of the All-Russian Collection of Biological Samples of Hereditary Diseases Biobank and were cryopreserved. RNA extraction was carried out using the Extract RNA reagent (Evrogen, Moscow, Russia). Reverse transcription was performed using the Reverse Transcription System (Dialat, Moscow, Russia) following the manufacturer’s protocol. The quality of the resulting cDNA was assessed by qPCR of the housekeeping gene B2M. To evaluate the variant’s effect on mRNA structure, the primer 5’-TGGCGTTCCTGGCAAGAG-3’ was designed to target the sequence of exon 12 of the *COL27A1* and 5’-CTGGGGGTCCCATCATGC-3’ was designed to target the sequence of exon 26. The primer annealing temperature was 60°C. The expected amplicon length is 731 bp. Following RT-PCR, deep targeted sequencing was performed, and the resulting reads were aligned to the hg38 reference genome. The alignment confirmed that the amplified locus was the *COL27A1* gene from exons 12 to 26, which includes the target locus of exons 16-19.

The NGS libraries were constructed using the "SG GM" Kit (Raissol) and sequenced on the FASTASeq platform in paired-end mode (2×150 bp). Sequencing depth exceeded 100000x for the proband, 30000x for the proband’s mother, and 50000x for control samples. Raw sequencing data were analyzed using a custom bioinformatics pipeline incorporating open-source tools. Initial quality assessment of reads was performed with FastQC (v0.12.1), followed by alignment to the hg38 reference genome and read sorting using STAR (v2.7.11b). Splice junction patterns were examined through Sashimi plots in IGV for visualization.

*Statistical analysis*

Descriptive statistical analysis was used to evaluate the frequency of clinical and radiological features. For each symptom, the relative frequency was calculated as the ratio of observed cases to the total number of patients assessed for that feature. To assess the statistical reliability of these frequencies, 95% confidence intervals were calculated using the Wilson score method. Data visualization was performed using Python 3.11 with NumPy, SciPy, and Matplotlib libraries. A forest plot was created to display point estimates and 95% CI for all features, with significant features (lower 95% CI > 50%) highlighted in a blue color.

**References:**

1. Beskorovainy N.S., Beskorovainaya T.S., Ryzhkova O.P., Shchoka E.V., Vizerov T.V., Stepanova A.A., Shchagina O.A., Polyakov A.V., Kutsev S.I. NGS-data-Omics. Certificate of State Registration of the Computer Program No. 2025685783 dated September 26, 2025.

2. Richards, S.; Aziz, N.; Bale, S.; Bick, D.; Das, S.; Gastier-Foster, J.; Grody, W.W.; Hegde, M.; Lyon, E.; Spector, E.; et al. Standards and guidelines for the interpretation of sequence variants: a joint consensus recommendation of the American College of Medical Genetics and Genomics and the Association for Molecular Pathology. Genet. Med. 2015, *17*, 405–424, doi:10.1038/GIM.2015.30.
