# Supplementary material for: Exploring the clinical and genetic spectrum of Steel syndrome: two case reports and review of the literature
Source: Front Med (Lausanne). 2026 Feb 18;13:1730466. doi: 10.3389/fmed.2026.1730466 (PMC12956644; doi:10.3389/fmed.2026.1730466)
Supplement: Supplementary file 2 [file Supplementary_file_2.docx]

Supplementary Material 2

***Supplementary Tables***

| **Table S1. Summary of clinical and radiological features of non-Puerto-Rican patients (part 1)** | | | | | | | | |
| --- | --- | --- | --- | --- | --- | --- | --- | --- |
| **Criteria** | **Kotabagi, 2010** | **Gariballa,**  **2017** | **Thuresson,**  **2018** | **Maddirevula, 2018** | **Pölsler, 2019** | **Kritioti,**  **2020** | **Gonzaga-**  **Jauregui, 2020**  **(BAB5133)** | **Gonzaga-Jauregui, 2020**  **(BAB10793)** |
| Number of patients | 1 | 1 | 1 | 1 | 1 | 1 | 1 | 1 |
| Ethnicity | Indian | Emirati | Iraqi | Yemeni | Syrian | Greek-Cypriot | Turkish | Turkish |
| Genotype | p.[Cys174SerfsTer34];  [Arg707Ter] | c.[3556-2A>G];  [3556-2A>G] | p.[Gly904Arg]; [Gly904Arg] | c.[4261-1G>A];  [4261-1G>A] | p.[Phe32LeufsTer71]; [Lys1026ArgfsTer33] | p.[Gly802Glu]; [Gly802Glu] | p.[Gly895Arg];  [Gly895Arg] | p.[Gly22SerfsTer6]; [Gly22SerfsTer6] |
| Sex | f | f | f | m | f | m | m | m |
| Age of last examination (y.o) | 5 | 3 | n/d | 5 | 9 | 4 | 6 | 1.8 |
| Short stature | + | + | n/d | n/d | +  85.5 cm (−7.9 SD) | +  96 cm (−2 SD) | + | + |
| Normal stature  (height more than  –2 SD) | – | – | n/d | n/d | – | – | – | – |
| Pectus excavatum | n/d | n/d | n/d | + | n/d | + | n/d | n/d |
| Limb shortening | n/d | +  (mild rhizomelic shortening of upper limbs) | +  (short upper limbs) | n/d | + | +  (mild mesomelic shortening) | n/d | +  (rhizomelic shortening of upper extremities) |
| **Facial features** | | | | | | | | |
| Oval-shaped face | + | – | n/d | + | + | – | + | n/d |
| Prominent forehead and/or frontal bossing | + | + | n/d | + | + | + | + | + |
| Hypertelorism | + | + | + | n/d | + | + | n/d | n/d |
| Midface hypoplasia | + | + | n/d | + | + | + | + | + |
| Long philtrum | + | + | n/d | (smooth) | + | + | + | + |
| **Radiological features** | | | | | | | | |
| Congenital hip dislocation | + | +  (unilateral) | + | + | + | n/d | + | + |
| Poorly ossified femoral head | + | + | n/d | n/d | n/d | + | + | + |
| Acetabular abnormalities | + | + | n/d | n/d | + | + | n/d | + |
| Carpal coalition | + | n/d | n/d | n/d | n/d | – | – | – |
| Radial head dislocation | + | +  (unilateral) | n/d | n/d | + | – | + | + |
| Scoliosis | +  (thoraco-lumbar) | n/d | +  (thoracal) | + | + | +  (mild thoracic) | – | – |
| Cervical spine abnormalities | n/d | + | n/d | n/d | n/d | n/d | n/d | n/d |
| Lumbar lordosis | + | n/d | n/d | n/d | + | – | –  (kyphosis) | + |
| Coxa vara | + | + | + | n/d | + | – | –  (coxa valga) | + |
| Genu valgum | + | + | n/d | n/d | n/d | + | + | + |
| Genu varum | – | – | n/d | n/d | n/d | – | – | – |
| Patellar dislocation | n/d | n/d | n/d | n/d | n/d | n/d | + | n/d |
| **Upper limbs abnormalities** | | | | | | | | |
| Cutaneous  syndactyly of fingers or/and toes | +  (partial) | n/d | n/d | n/d | n/d | + | n/d | n/d |
| Bilateral 5^th^ finger clinodactyly | + | n/d | n/d | n/d | n/d | + | + | + |
| **Lower limbs abnormalities** | | | | | | | | |
| Various foot deformity | +  clubfoot | n/d | pes cavus | n/d | n/d | – | pes equinovarus  pes planovalgus | unspecified mild foot deformity |
| Vertical talus | + | n/d | n/d | n/d | + | – | n/d | n/d |
| **Extraskeletal features** | | | | | | | | |
| Cryptorchidism | n/a | n/a | n/a | + | n/a | n/a | n/d | n/d |
| Inguinal or umblical hernia | n/d | n/d | n/d | +  (inguinal) | n/d | n/d | n/d | n/d |
| Developmental delay | + | n/d | +  (mild) | + | n/d | _ | +  (mild) | + |
| Hearing loss | + | +  SNHL  (at 2.5 y) | + | n/d | +  SNHL (at 3.8 y) | +  SNHL | +  (conductive) | + |
| Motor delay | + | – | n/d | n/d | + | – | n/d | n/d |
| Delayed speech | + | + | n/d | n/d | + | + | n/d | n/d |
| Other | right sided hydronephrosis with lower pole caliectasis | contractures at the right elbow; mild bowing of the femur; fusion at the posterior aspect of the spinous processes of C3 and C4; with increased height of the intervertebral disk with slight dislocation between C4 and C5 | tethered spinal cord | – | bilateral inferior nasal iris colobomata and bilateral choroido-retinal colobomata | short mid phalanges,  external rotation of right foot | conductive hearing loss due to frequent respiratory and ear infections during the neonatal period;  torticollis;  lumbar kyphosis;  hyperlaxity;  hypoplastic clavicles; ventricular septal defect;  femoral bowing | femoral bowing; rhizomelic shortening of upper extremities; walking difficulties; pointing long vertebral pedicles; tall vertebral bodies, long vertebral pedicles |

n/d – no data; n/a – not applicable

**Table S2. Summary of clinical and radiological features of non-Puerto-Rican patients (part 2)**

| **Criteria** | **Gonzaga-Jauregui, 2020  (BAB8900)** | **Satoh, 2021** | **Kim, 2021** | **Girisha, 2022** | **Girisha, 2022** |
| --- | --- | --- | --- | --- | --- |
| Number of patients | 1 | 2 | 1 | 2 | 1 |
| Ethnicity | Turkish | Japanese | Korean | Indian | Indian |
| Genotype | p.[Gly1660AspfsTer3];  [Gly1660AspfsTer3] | p.[Gly676Arg]; [Met788ValfsTer34] | p.[Gly1412ArgfsTer157];  [Gly1240_Lys1812del] | p.[Ala99Thr];  [Pro1019His] | p.[Gly841Arg];  [Gly841Arg] |
| Sex | f | m;m (sibs) | m | m;m  (monozygotic twins) | m |
| Age of last examination (y.o) | 11 | 5.8; 3.7 | 11 | 6 | 1.4 |
| Short stature | + | – | +  120.5 cm (-3.2 SD) | 2/2  95 cm (-4.4 SD)  94 cm (-4.6 SD) | +  68 cm (-4 SD) |
| Normal stature  (height more than –2 SD) | – | 2/2  105.3 cm (-1.26 SD)  93 cm (-0.97 SD) | – | – | – |
| Pectus excavatum | n/d | n/d | + | 2/2 | + |
| Limb shortening | +  (rhizomelic shortening of upper extremities) | 2/2  (rhizomelia) | + | – | – |
| **Facial features** | | | | | |
| Oval-shaped face | + | – | n/d | 2/2 | + |
| Prominent forehead and/or frontal bossing | – | 2/2 | + | 2/2 | + |
| Hypertelorism | n/d | – | + | 2/2 | + |
| Midface hypoplasia | + | 2/2 | + | 2/2 | + |
| Long philtrum | + | n/d | n/d | 2/2 | – |
| **Radiological features** | | | | | |
| Congenital hip dislocation | + | 2/2 | – | 2/2 (subluxation) | + |
| Poorly ossified femoral head | n/d | n/d | + | 2/2 | + |
| Acetabular abnormalities | + | n/d | + | 2/2 | + |
| Carpal coalition | + | – | + | – | – |
| Radial head dislocation | + | 2/2 | + | 2/2 | + |
| Scoliosis | + | – | +  (mild thoracolumbar kyphoscoliosis) | 1/2 | – |
| Cervical spine abnormalities | n/d | n/d | – | – | – |
| Lumbar lordosis | + | n/d | – | – | – |
| Coxa vara | n/d | n/d | + | 2/2 | – |
| Genu valgum | + | 2/2 | + | – | – |
| Genu varum | – | – | – | 2/2 | + |
| Patellar dislocation | + | 2/2 | + | n/d | n/d |
| **Upper limbs abnormalities** | | | | | |
| Cutaneous  syndactyly of  fingers or/and toes | n/d | 2/2 | n/d | 2/2  (partial) | +  (partial) |
| Bilateral 5^th^ finger clinodactyly | + | 2/2 | n/d | 2/2 | + |
| **Lower limbs abnormalities** | | | | | |
| Various foot deformity | Pes equinovarus  Pes cavus | (n=1) left talipes valgus, right vertical talus  (n=1) bilateral talipes valgus | n/d | – | calcaneovalgus of both feet  pes planus |
| Vertical talus | + | 2/2 | n/d | n/d | n/d |
| **Extraskeletal features** | | | | | |
| Cryptorchidism | n/a | 1/2 | + | 2/2 | + |
| Inguinal or umblical hernia | n/d | umblical hernia (n=1) | n/d | 2/2 | + |
| Developmental delay | + | 2/2 | – | – | – |
| Hearing loss | + | 2/2 | + | 2/2 | + SNHL |
| Motor delay | n/d | + | – | – | – |
| Delayed speech | n/d | 2/2 | – | – | – |
| Other | limited elbow and  hip movement, walking difficulties; tarsal coalitions; tall vertebral bodies; partial knee  dislocation (right worse than left) and pes cavu | atrial septal defect  micrognathia,crowded  teeth, small penis (n=2) | hypoplastic vertebral body of L1  mild prognathism  flexion contracture of the left elbow | delayed ossification of carpal bones  cleft palate  retrognathia | atrial septal defect  retrognathia |

n/d – no data; n/a – not applicable

**Table S3. Summary of clinical and radiological features of Puerto-Rican patients**

| **Criteria** | **Gonzaga-Jauregui, 2015** | **Belbin, 2017** | **Amlie-Wolf, 2020** | **Gonzaga-Jauregui, 2020**  **(STLS patients)** | **^#^Gonzaga-Jauregui, 2020**  **(Family HOU2809)** | **Gonzaga-Jauregui, 2020**  **(GHS01)** |
| --- | --- | --- | --- | --- | --- | --- |
| Number of patients | 2 | 5 | 3 | 5 | 3 | 1 |
| Ethnicity | Puerto Rican | | | | | Hispanic |
| Genotype | p.[Gly697Arg];[Gly697Arg] | | | | | |
| Sex | f;m (sibs) | f (n=3); m (n=2) | f (n=1); m (n=2) | f (n=2); m (n=3) | m;f;f | f |
| Age of last examination (years) | 12; 14 | mean age 51.6 (34-74) | 0.16 ;7; 14 | n/d | 33;39;48 | 39 |
| Short stature | 2/2 | 5/5 | 1/3  110.5 cm  (-2.1 SD) | 5/5 | 3/3  157 cm (‐2.75 SD)  147 cm (‐2.46 SD)  140 cm (‐3.58 SD) | + |
| Normal stature  (more than –2 SD) | – | – | 2/3  153 cm  (-1.3 SD)  73.6 cm  (-1.57 SD) | – | – | – |
| Pectus excavatum | n/d | n/d | 2/3 | n/d | n/d | n/d |
| Limb shortening | n/d | n/d | n/d | n/d | n/d | n/d |
| **Facial features** | | | | | | |
| Oval-shaped face | 2/2 | n/d | n/d | n/d | n/d | n/d |
| Prominent forehead and/or frontal bossing | 2/2 | n/d | 1/3 | n/d | n/d | n/d |
| Hypertelorism | 2/2 | n/d | 1/3 | n/d | + | n/d |
| Midface hypoplasia | 2/2 | n/d | 2/3 | n/d | n/d | n/d |
| Long philtrum | 2/2 | n/d | n/d | n/d | n/d | n/d |
| **Radiological features** | | | | | | |
| Congenital hip dislocation | 2/2 | – | 3/3 | 5/5 | 3/3 | + |
| Poorly ossified femoral head | 2/2 | n/d | 1/3 | n/d | n/d | n/d |
| Acetabular abnormalities | 2/2 | n/d | 1/3 | n/d | + | n/d |
| Carpal coalition | 2/2 | 2/5 | n/d | 5/5 | n/d | n/d |
| Radial head dislocation | 1/2 | 1/5 | 2/3 | 5/5 | 3/3 | + |
| Scoliosis | 2/2  (thoracic scoliosis) | 2/5 | 2/3 | 5/5 | 3/3 | + |
| Cervical spine abnormalities | 1/2  odontoid hypoplasia | cervical stenosis (5/5)  cervical cord compression (3/5) | n/d | n/d | n/d | +  (cervical  radiculopathy) |
| Lumbar lordosis | 1/2 | 1/5 | n/d | + | 3/3 | n/d |
| Coxa vara | 1/2 | n/d | n/d | n/d | n/d | n/d |
| Genu valgum | n/d | n/d | n/d | n/d | + | n/d |
| Genu varum | n/d | n/d | n/d | n/d | n/d | n/d |
| Patellar dislocation | n/d | n/d | n/d | n/d | + | n/d |
| **Upper limbs abnormalities** | | | | | | |
| Partial cutaneous  syndactyly of  fingers or/and toes | n/d | n/d | n/d | n/d | n/d | n/d |
| Bilateral 5^th^ finger clinodactyly | 2/2 | n/d | n/d | n/d | n/d | n/d |
| **Lower limbs abnormalities** | | | | | | |
| Various foot deformity | pes planus  2/2 | – | metatarsus adductus (1/3) | n/d | displacement of the cuneiform bone and pes planus (n=1) | +  (unspecified deformation) |
| Vertical talus | – | n/d | n/d | n/d | n/d | n/d |
| **Extraskeletal features** | | | | | | |
| Cryptorchidism | n/d | n/d | n/d | n/d | n/d | n/a |
| Inguinal or umblical hernia | n/d | n/d | n/d | n/d | n/d | n/d |
| Developmental delay | – | n/d | 1/3 | n/d | – | n/d |
| Hearing loss | – | n/d | – | n/d | 3/3 (adult‐onset) | +SNHL |
| Motor delay | 1/2 | n/d | 2/3 | n/d | + | n/d |
| Delayed speech | n/d | n/d | n/d | n/d | n/d | n/d |
| Other | bilateral decreased elbow extension | knee replacement (n=2)  Hip replacement (n=3) | 1/3 hip dysplasia, microcephaly, laryngomalacia, oromotor dysphagia, muscle weekness, mild hypotonia, ligamentous laxity  1/3 bony fusion involving the calcaneus and lateral process of the talus of the ankle  1/3 hypermobility in hands and toes with restricted extension at both elbows | – | numerous joint dislocations  involving the knees, elbows, and toes (BAB7802);  joint laxity with recurrent joint dislocations, hypothyroidism, Chiari  malformation, and a tethered cord (BAB9002);  joint laxity with recurrent joint dislocation (BAB9010) | leg length discrepancy;  failure of total hip arthroplasty |

^#^It is not specified in the article whether some features are present or absent in the patients. Consequently, these features were excluded from the overall analysis summarized in Table 1.

n/d – no data; n/a – not applicable

**Table S4. Summary of reported nucleotide variants identified in *COL27A1***

| **№** | **HGVS_Genomic_GRCh38** | **HGVS_transcript** | **HGVS_Predicted_Protein** | **Type of SNVs** | **References** |
| --- | --- | --- | --- | --- | --- |
| 1 | chr9:114162715_114162721del | NM_032888.4:c.63_69del | p.Gly22SerfsTer6 | frameshift | Gonzaga-Jauregui, 2020 (BAB10793) |
| 2 | chr9:114162745del | NM_032888.4:c.93del | p.Phe32LeufsTer71 | frameshift | Pölsler, 2019 |
| 3 | chr9:114167850G>A | NM_032888.4:c.295G>A | p.Ala99Thr | missense | Girisha, 2022 |
| 4 | chr9:114168076_114168083del | NM_032888.4:c.521_528del | p.Cys174SerfsTer34 | frameshift | Kotabagi, 2010 |
| 5 | chr9:114194413G>C | NM_032888.4:c.2026G>C | p.Gly676Arg | missense | Satoh, 2021 |
| 6 | chr9:114195977G>C | NM_032888.4:c.2089G>C | p.Gly697Arg | missense | Gonzaga-Jauregui, 2015  Belbin, 2017  Amlie-Wolf, 2020  Gonzaga-Jauregui, 2020 |
| 7 | chr9:114196007C>T | NM_032888.4:c.2119C>T | p.Arg707Ter | nonsense | Kotabagi, 2010 |
| 8^#^ | chr9:114211026G>A | NM_032888.4:c.2367G>A | p.Met788ValfsTer34 | frameshift | Satoh, 2021 |
| 9 | chr9:114219828G>A | NM_032888.4:c.2405G>A | p.Gly802Glu | missense | Kritioti, 2020 |
| 10 | chr9:114231822G>A | NM_032888.4:c.2521G>A | p.Gly841Arg | missense | Girisha, 2022 |
| 11 | chr9:114231849G>A | NM_032888.4:c.2548G>A | p.Gly850Arg | missense | Frigola, 2021 |
| 12* | chr9:114235653G>A | NM_032888.4:c.2619+1G>A | p.Gly856_Lys873del | (splice)  in-frame deletion | present study |
| 13* | chr9:114237038A>G | NM_032888.4:c.2673+4A>G | p.Gly874_Leu891del | (splice)  in-frame deletion | present study |
| 14 | chr9:114237671G>A | NM_032888.4:c.2683G>A | p.Gly895Arg | missense | Gonzaga- Jauregui, 2020  (BAB5133) |
| 15 | chr9:114237698G>A | NM_032888.4:c.2710G>A | p.Gly904Arg | missense | Thuresson, 2018 |
| 16 | chr9:114252615C>A | NM_032888.4:c.3056C>A | p.Pro1019His | missense | Girisha, 2022 |
| 17 | chr9:114252634del | NM_032888.4:c.3075del | p.Lys1026ArgfsTer33 | frameshift | Pölsler, 2019 |
| 18 | chr9:114264409G>T | NM_032888.4:c.3249+1G>T | p.? | splice | Frigola, 2021 |
| 19 | chr9:114270726A>G | NM_032888.4:c.3556-2A>G | p.? | splice | Gariballa, 2017 |
| 20 | chr9:114282278_114309479del | NM_032888.4:c.3718_5436del | p.Gly1240_Lys1812del | in-frame deletion | Kim, 2021 |
| 21* | chr9:114288455G>C | NM_032888.4:c.3988G>C | p.Gly1330Arg | missense | present study |
| 22 | chr9:114290223G>A | NM_032888.4:c.4261-1G>A | p.? | splice | Maddirevula, 2018 |
| 23 | chr9:114290080_114290084dup | NM_032888.4:c.4229_4233dup | p.Gly1412ArgfsTer157 | frameshift | Kim, 2021 |
| 24 | chr9:114306560_114306564del | NM_032888.4:c.4979_4983del | p.Gly1660AspfsTer3 | frameshift | Gonzaga-Jauregui, 2020  (BAB8900) |

* Nucleotide variants №12, №13 and №21 were identified in present study

^#^ Nucleotide variant was annotated according RNA analysis results performed by Satoh, 2021

***Supplementary Figures***

**Figure S1. Analysis of phenotypic feature frequencies in reported patients with STLS, including those from the present study (n = 63)**


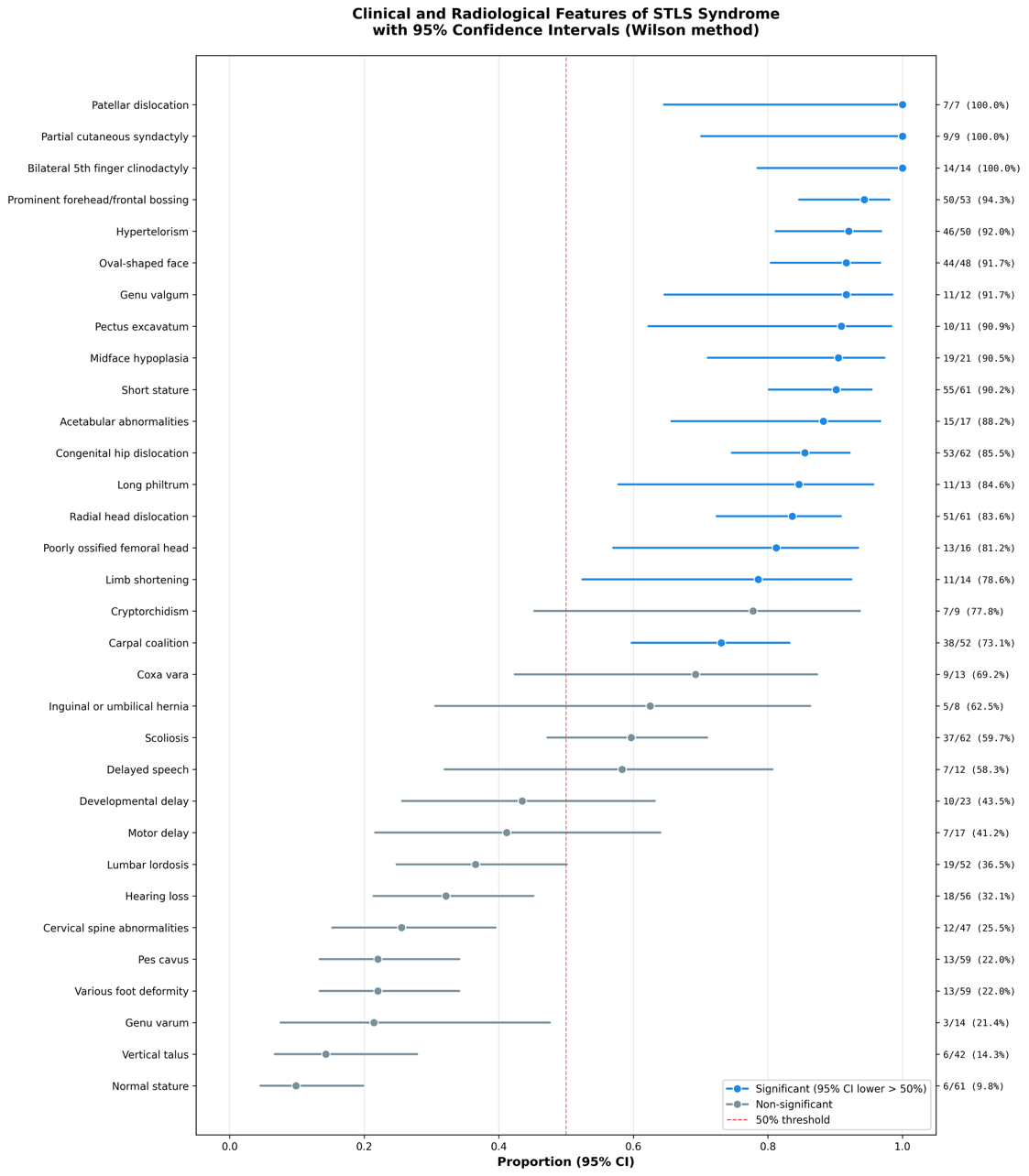


The most frequently observed features with narrow CI included short stature (55/61), congenital hip dislocation (53/62), and radial head dislocation (51/61), as well as characteristic craniofacial features: prominent forehead/frontal bossing (50/53), hypertelorism (46/50), and an oval-shaped face (44/48). Among radiographic abnormalities carpal coalition was also commonly reported (38/52), supporting the consideration of these findings as key features of Steel syndrome.

It is important to note that patellar dislocation (7/7), partial cutaneous syndactyly (9/9), and bilateral fifth-finger clinodactyly (14/14) were indeed reported in all patients in whom these features were assessed or described. Therefore, they appear as frequent on the plot. However, the number of observations for these features is small, resulting in wide CIs, meaning that their true prevalence remains uncertain. Accordingly, these findings should be interpreted with caution.

**Figure S2. Sanger sequencing data from the two families in the present study.**


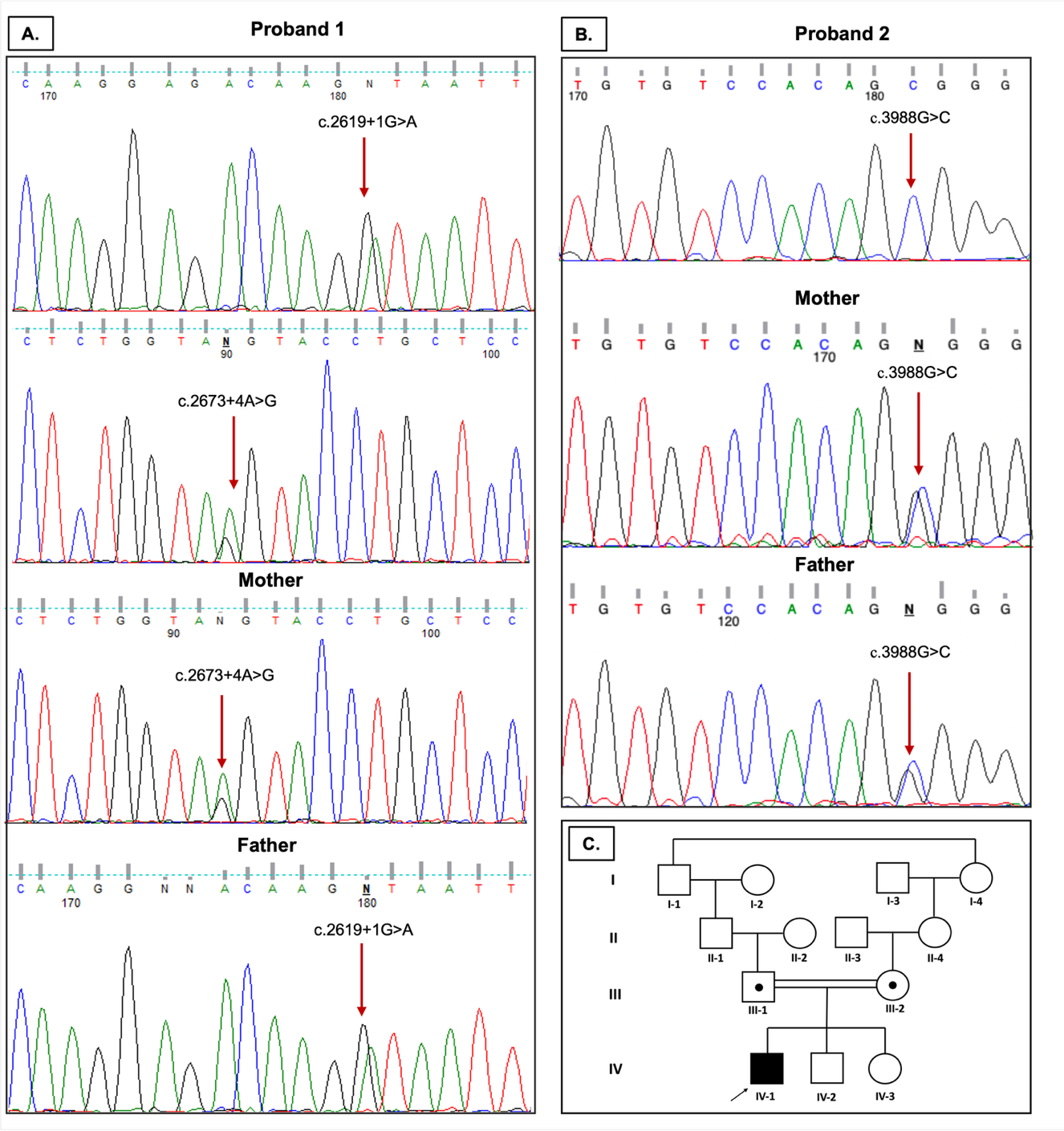


1. **For Family of the P1,** Sanger sequencing confirmed compound heterozygosity for the ***COL27A1*** variants c.2619+1G>A and c.2673+4A>G.
2. **For Family of the P2,** Sanger sequencing confirmed homozygosity for the ***COL27A1*** variant c.3988G>C p.(Gly1330Arg).
3. Pedigree of the P2 family.

**References:**

1. Kotabagi, S.; Shah, H.; Shukla, A.; Girisha, K.M. Second family provides further evidence for causation of Steel syndrome by biallelic mutations in COL27A1. *Clin. Genet.* **2017**, *92*, 323–326, doi:10.1111/CGE.13006.
2. Gariballa, N.; Ben-Mahmoud, A.; Komara, M.; Al-Shamsi, A.M.; John, A.; Ali, B.R.; Al-Gazali, L. A novel aberrant splice site mutation in COL27A1 is responsible for Steel syndrome and extension of the phenotype to include hearing loss. *Am. J. Med. Genet. Part A* **2017**, *173*, 1257–1263, doi:10.1002/AJMG.A.38153.
3. Thuresson, A.C.; Soussi Zander, C.; Zhao, J.J.; Halvardson, J.; Maqbool, K.; Månsson, E.; Stenninger, E.; Holmlund, U.; Öhrner, Y.; Feuk, L. Whole genome sequencing of consanguineous families reveals novel pathogenic variants in intellectual disability. *Clin. Genet.* **2019**, *95*, 436–439, doi:10.1111/CGE.13470/.
4. Maddirevula, S.; Alzahrani, F.; Al-Owain, M.; Al Muhaizea, M.A.; Kayyali, H.R.; AlHashem, A.; Rahbeeni, Z.; Al-Otaibi, M.; Alzaidan, H.I.; Balobaid, A.; et al. Autozygome and high throughput confirmation of disease genes candidacy. *Genet. Med.* **2019**, *21*, 736–742, doi:10.1038/s41436-018-0138-x.
5. Pölsler, L.; Schatz, U.A.; Simma, B.; Zschocke, J.; Rudnik-Schöneborn, S. A Syrian patient with Steel syndrome due to compound heterozygous COL27A1 mutations with colobomata of the eye. *Am. J. Med. Genet. Part A* **2020**, *182*, 730–734, doi:10.1002/AJMG.A.61478.
6. Evie Kritioti; Athina Theodosiou; Nayia Nicolaou; Angelos Alexandrou; Ioannis Papaevripidou; Elisavet Efstathiou; Violetta Christophidou-Anastasiadou; Carolina Sismani; Tanteles, G.A. First reported case of Steel syndrome in the European population: A novel homozygous mutation in COL27A1 and review of the literature. Eur. J. Med. Genet. 2020, 63, doi:10.1016/j.ejmg.2020.103939.
7. Gonzaga-Jauregui, C.; Yesil, G.; Nistala, H.; Gezdirici, A.; Bayram, Y.; Nannuru, K.C.; Pehlivan, D.; Yuan, B.; Jimenez, J.; Sahin, Y.; et al. Functional biology of the Steel syndrome founder allele and evidence for clan genomics derivation of COL27A1 pathogenic alleles worldwide. *Eur. J. Hum. Genet. 2020 289* **2020**, *28*, 1243–1264, doi:10.1038/s41431-020-0632-x.
8. Satoh, C.; Kondoh, T.; Shimizu, H.; Kinoshita, A.; Mishima, H.; Nishimura, G.; Miyazaki, M.; Okano, K.; Kumai, Y.; Yoshiura, K. ichiro Brothers with novel compound heterozygous mutations in COL27A1 causing dental and genital abnormalities. *Eur. J. Med. Genet.* **2021**, *64*, doi:10.1016/J.EJMG.2020.104125.
9. Kim, J.S.; Jeon, H.; Lee, H.; Ko, J.M.; Kim, Y.; Choi, M.; Nishimura, G.; Kim, O.H.; Cho, T.J. Biallelic novel mutations of the COL27A1 gene in a patient with Steel syndrome. *Hum. Genome Var.* **2021**, *8*, 1–4, doi:10.1038/s41439-021-00149-7.
10. Girisha, K.M.; Jacob, P.; SriLakshmi Bhavani, G.; Shah, H.; Mortier, G.R. Steel syndrome: Report of three patients, including monozygotic twins and review of clinical and mutation profiles. Eur. J. Med. Genet. 2022, 65, doi:10.1016/j.ejmg.2022.104521.
11. Gonzaga-Jauregui, C.; Gamble, C.N.; Yuan, B.; Penney, S.; Jhangiani, S.; Muzny, D.M.; Gibbs, R.A.; Lupski, J.R.; Hecht, J.T. Mutations in COL27A1 cause Steel syndrome and suggest a founder mutation effect in the Puerto Rican population. *Eur. J. Hum. Genet.* **2015**, *23*, 342–346, doi:10.1038/ejhg.2014.107.
12. Belbin, G.M.; Odgis, J.; Sorokin, E.P.; Yee, M.C.; Kohli, S.; Glicksberg, B.S.; Gignoux, C.R.; Wojcik, G.L.; Van Vleck, T.; Jeff, J.M.; et al. Genetic identification of a common collagen disease in puerto ricans via identity-by-descent mapping in a health system. *Elife* **2017**, *6*, doi:10.7554/ELIFE.25060.
13. Amlie-Wolf, L.; Moyer-Harasink, S.; Carr, A.M.; Giampietro, P.; Schneider, A.; Simon, M. Three new patients with Steel syndrome and a Puerto Rican specific COL27A1 mutation. Am. J. Med. Genet. Part A 2020, 182, 798–803, doi:10.1002/AJMG.A.61465.
